# Development and validation of measurement tools for user experience evaluation surveys in the public primary healthcare facilities in Greece: a mixed methods study

**DOI:** 10.1186/s12875-019-0935-6

**Published:** 2019-04-02

**Authors:** Daphne Kaitelidou, Charalambos Economou, Petros Galanis, Olympia Konstantakopoulou, Olga Siskou, Silviu Domente, Dolf de Boer, Wienke G. Boerma, Peter P. Groenewegen

**Affiliations:** 1 Centre for Health Services Management and Evaluation (CHESME), National and Kapodistrian University of Athens (NKUA), Faculty of Nursing, Athens, Greece; 2 Panteion University of Athens, Athens, Greece; 3 Adjunct Faculty Member (Tutor) on Open University of Cyprus (OUC), Centre for Health Services Management and Evaluation (CHESME), National and Kapodistrian University of Athens (NKUA), Faculty of Nursing, Athens, Greece; 4 WHO Europe Regional Office, Athens, Greece; 5 Netherlands Institute for Health Services research (NIVEL), Utrecht, The Netherlands; 6 Department of Sociology, Department of Human Geography Utrecht University, Utrecht, The Netherlands

**Keywords:** Primary healthcare, Greece, Measurement tool, Users’ experience, Validation

## Abstract

**Background:**

The public primary healthcare system in Greece has not been fully developed and is delivered by urban and rural health centers, outpatient departments in public hospitals and the recently established first-contact and decentralized local primary care units. The aim of this study was to develop a valid and reliable measurement tool for conducting periodic user experience evaluation surveys in public Primary HealthCare facilities in Greece such as outpatient clinics of public hospitals and health centers.

**Methods:**

A mixed methods approach was applied. In particular, the methodology of developing and validating the tools included three steps: (a) establishment of the theoretical background/literature review, (b) qualitative study: development of the tools items and establishment of the face validity and (c) quantitative study: pilot testing and establishment of the structural validity and estimation of the internal consistency of the tools. Two patient focus groups participated in qualitative study: one visiting health centres and the other visiting the outpatient clinics of public hospitals. Quantitative study included 733 Primary Health Care services’ users/patients and was conducted during August–October 2017. Exploratory and confirmatory factor analysis was performed to check for structural validity of the tools, while Cronbach’s alpha coefficients were estimated to check for reliability.

**Results:**

Confirmatory factor analysis confirmed almost perfectly the presumed theoretical model and the following six factors were identified through the tools: (a) accessibility (three items, e.g. opening hours), (b) continuity and coordination of care (three items, e.g. doctor asks for medical history), (c) comprehensiveness of care (three items, e.g. doctor provides advices for healthy life), (d) quality of medical care (four items, e.g. sufficient examination time), (e) facility (four items, e.g. comfortable waiting room) and (f) quality of care provided by nurses and other health professionals (four items, e.g. polite nurses).

**Conclusions:**

We have developed reliable and valid tools to measure users’ experiences in public Primary HealthCare facilities in Greece. These tools could be very useful in examining differences between different types of public Primary Health Care facilities and different populations.

**Electronic supplementary material:**

The online version of this article (10.1186/s12875-019-0935-6) contains supplementary material, which is available to authorized users.

## Background

According to the key elements set by WHO on achieving the Primary Health Care’s (PHC) ultimate goal “Better health for all”, service delivery reforms in terms of “organizing health services around people’s needs and expectations” stands high on the list of its priorities [1]. Reinforcing the PHC system of a country can both improve health outcomes and reduce unnecessary costs [2, 3]. This is especially relevant for Greece as, since 2010, the Greek economy has undergone a deep fiscal and structural economic crisis. This has also affected the health care sector. PHC has been brought forward, although with considerable delay, as the most prominent strategy for improving health status and quality of healthcare services, while, at the same time, streamlining costs and reducing the burden on the government expenditure [4]. Given the aim of organizing health services around people’s needs and expectations, it is important to monitor and evaluate the policy implementation from the point of view of the users of health services.

In recent years, the interest of both health policy and research stakeholders has centered upon healthcare users’ experiences in order to monitor and evaluate the implementation of patient-centered services and whether these fulfill their needs, preferences and values [5, 6]. In the USA, the evaluation of the quality of ambulatory health services for the largest insurance funds has been conducted through surveys and protocols of the CAHPS (Consumer Assessment of Healthcare Providers and Systems) and HEDIS (Healthcare Effectiveness and Data Information Set) [7]. These methodological approaches are based on user experiences and take into account medical effectiveness, interpersonal relationships, accessibility, cost and other parameters [8]. In Europe, the QUALICOPC (Quality and Costs of Primary Care in Europe) study aimed at assessing primary health care from the perspective of its users in more than 30 countries, including Greece [9–11].

Although patient satisfaction has been used towards assessing the different dimensions of health care [12, 13], designing effective health care management strategies [14] and redesigning the goals of health services management within the framework of improving their quality, patients’ experiences constitute one of the most essential components of the evaluation of the quality of the health care system and services [15]. The concept of recording healthcare services users’ experiences refers to their “journey” as a whole, within the health system [16]. The monitoring and evaluation of their experiences, with administrative and clinical aspects of healthcare services, is important since it provides information from the point of view of users that helps stakeholders to improve quality.

Experiences are far more proximal to objective observations than satisfaction, since the latter being much more proximal to subjective evaluations. In particular, patients may be in a physical or psychological distress that does not allow them to have an objective view of satisfaction. The same applies in the cases of rapid rotation of interventions, diagnostic procedures and measurements, as it does not make it easier for them to form a complete picture. In addition, the concept of quality and satisfaction is related to cultural habits; therefore it varies from place to place and is also influenced by characteristics such as age, gender, educational level and economic situation, which are not directly related to the healthcare services provided [17–20]. Satisfaction surveys may be misleading, especially if patients do not have the opportunity to comment objectively and in detail on specific aspects of the services they have received and their experience as a whole. It has also been found that in such surveys, where participants are asked to report on how happy they are, they often hesitate to respond or tend to respond positively [21]. This is mainly due to the fact that the questions asked are subjective and require from participants to make value and/or emotional criticism, which is influenced to a large extent by factors such as their expectations and preferences [17, 22].

The public primary health care system in Greece has not been fully developed and is delivered by urban and rural health centres, outpatient departments in public hospitals and the recently established first-contact and decentralized local primary care units (TOMYs) (65 TOMYs have been set up so far – May 2017). We did not identify a national “tailor-made” tool for assessing the experiences of the users at primary health care level in Greece and as the country is now in the process of this important reform regarding the PHC sector, a development of such a tool has been an imperative need.

The aim of this study was to develop a valid and reliable measurement tool for conducting periodic user experience evaluation surveys in public Primary HealthCare facilities in Greece such as outpatient clinics of public hospitals and health centers.

## Methods

**Fig. 1:**
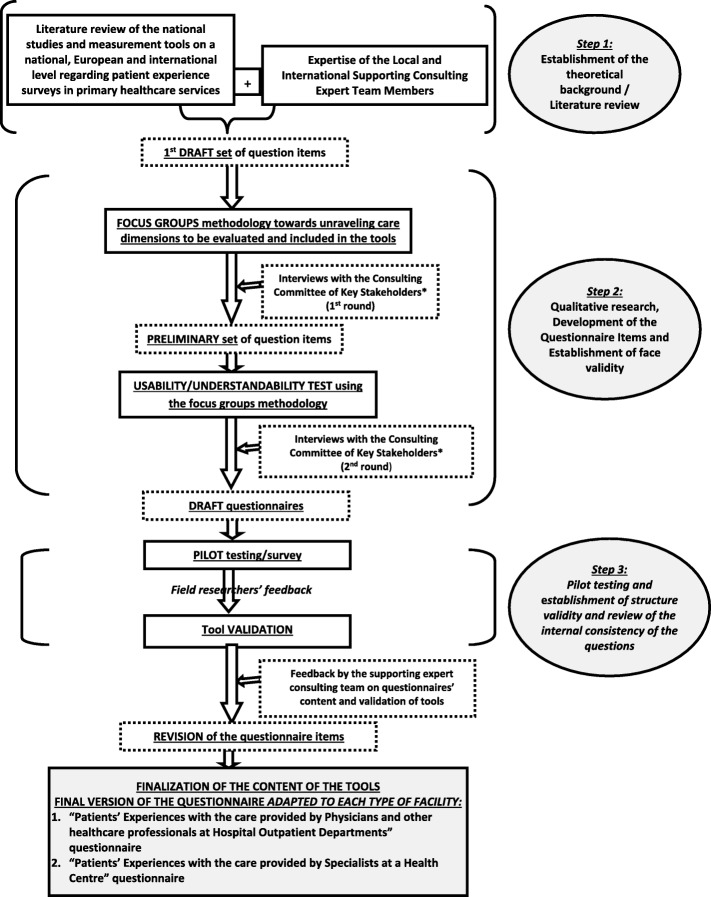
Steps in developing and validating tools for conducting periodic user

The aim of this study was to develop a valid and reliable measurement tool for conducting periodic user experience evaluation surveys in public Primary Health Care facilities in Greece such as outpatient clinics of public hospitals and health centers. A mixed methods approach was applied. In particular, the methodology of developing and validating the tools included three steps: (a) establishment of the theoretical background/literature review, (b) qualitative study: development of the tools items and establishment of the face validity and (c) quantitative study: pilot testing and establishment of the structural validity and estimation of the internal consistency of the tools (Fig. 1).

### Step 1: establishment of the theoretical background/literature review

In order to establish a theoretical background on the evaluated dimensions of care in primary healthcare services, a literature review of the international as well as the national studies regarding patient experience surveys was conducted initially, focusing on the tools used and the reported evaluated dimensions of care in the PHC sector. Search was conducted in PubMed and Scopus using keywords such as patient(s), experience(s), healthcare service(s), Greece, Greek, healthcare, quality, primary care unit(s), primary healthcare, primary healthcare service(s), perception(s), opinion(s) etc., with emphasis on the papers published during the last decade. National journals published in Greek as well as grey national literature were also considered for relevant reports or papers. This step resulted in the identification of the most widely used measurement tools (on the national, European and International level) and of the dimensions for measuring primary health care. Additionally, the authors (i.e. the local and international supporting consulting expert team members) contributed with their expertise on the PHC sector and development of measurement tools. Thus, a first draft set of question items was developed per type of facility and per dimension of care evaluated.

### Step 2: qualitative study: development of the questionnaire items and establishment of face validity

The First draft set of question items were presented to two patient focus groups (one visiting health centres and the other visiting the outpatient clinics of public hospitals) in order to unravel care dimensions and items to be evaluated (and ultimately to be included in the tools), ensure the inclusion of any missing aspects and contribute to the usability/understandability of the questions [23]. Patients reviewed the first set of measurement items developed during this step, commented on their appropriateness and added to the existing items. Their feedback was appropriately processed and analyzed, resulting to a preliminary set of questions. During step 2, a consulting committee of key stakeholders familiar with the PHC sector (Health professionals, Patient representatives, Health policy makers) was installed and a list with the pilot locations was compiled. The consulting committee consisted of 10 health professionals and policy makers either working in primary care settings or being affiliated with central governmental primary health care bodies as well as patient representatives. Among others, the consulting committee contributed to identifying the intended goals that the survey would be designed to meet as well as to participate in the face validity process of the questionnaire.

The process of ensuring face validity was initiated by having the first draft set of question items (developed during step 1) reviewed by the key groups of stakeholders; a first round of interviews was held and stakeholders evaluated whether the questions successfully captured the wide spectrum of the PHC dimensions, commented on the questionnaires’ content and construction, contributing to the survey not containing common errors such as leading, confusing or double-barreled questions.

Afterwards, these pre-final versions of the questionnaire were drafted and a usability/understandability test was conducted using the focus groups methodology. The usability test altered the content and structure of the questionnaire and this revised draft questionnaire (adapted to each type of PHC facility) was provided to the Consulting Committee of Key Stakeholders for a second round of interviews. As a result, the draft questionnaire to be administered in the pilot primary health care facilities was finalised.

### Step 3: quantitative study: pilot testing, establishment of structural validity and estimation of internal consistency

#### Pilot testing

In step 3, the final self–completed draft questionnaire was administered to a study population of 733 PHC services’ users/patients who visited a selected subset of PHC facilities (8 Public GP services’ practices/Health centers and 3 Outpatient clinics of Public Hospitals) during August–October 2017 in order to field-test the tool’s content, usability and appropriateness for the target population and PHC facilities. At this point, the field researchers’ experiences during this pilot phase were considered to be vital so as to identify comprehension strains on site and during the completion of the questionnaires and highlight any questionnaire items that were difficult to understand and generated misinterpretations; besides, the feedback received by the field researchers along with the results of the validation process and pilot testing constituted the basis for the development of the final version of the questionnaires.

Convenience sampling was used so as for the study sample to be drawn. Service users were recruited just after having concluded their consultation with the healthcare professional upon leaving the examination room. There were no other criteria to the sampling method except that the users/patients were available and willing to participate. Field researchers informed the potential participants in detail regarding the aim of the survey and their informed consent was sought so as to enroll a PHC user to the study. The sample was drawn from patients or their adult companion who received care from a primary health care unit and the inclusion criteria were consenting adults 18 years or older, with ease of reading and writing in the Greek language. Participants with physical disabilities that might interfere with their ability to give informed consent, cooperate with the staff of PHC facilities, or understand the questions asked were facilitated by the field researchers towards the completion of the questionnaire.

#### Establishment of structure validity and estimation of the internal consistency

Exploratory and confirmatory factor analyses were conducted so as to identify the underlying components/factors (i.e. dimensions of care); questions that pointed back to the same dimensions should have loaded into the same factors. Finally, the internal consistency of the questions that loaded onto the same factors was checked using the Cronbach’s alpha coefficient and by checking the correlation between questions that load on the same factor so as to ensure the survey answers were consistent.

The final stage of the validation process was to revise the survey/questionnaire items based on the information gathered from the factor analysis and Cronbach’s alpha coefficient. Minor changes were made based on these results and the theoretical background formulated during step 1 regarding the evaluated dimensions of care in primary healthcare services. Following these revisions, the final content of the tools for conducting periodic user experience evaluation surveys in primary public health care facilities was formulated and finalised.

### Ethical issues

Participants were orally and in written informed about the purpose and methodology of the study so as to decide whether or not they were willing to participate voluntarily and anonymously. Signed consent forms were delivered to the researchers prior to the completion of the questionnaires. Approvals of the research protocol were ensured by the Scientific Committees of the selected public hospitals, the CEOs of the Health Regions supervising the selected Health Centers and the Ministry of Health. The study was supported by the WHO Regional Office for Europe.

### Statistical analysis

In order to check for structural validity and reliability of the questionnaires, factor analysis was conducted so as for independent latent variables to be unraveled and Cronbach’s alpha coefficients were accordingly calculated. First, we performed an exploratory, varimax rotation, principal components factor analysis to determine the factor structure of the questionnaires. With regard to the number of factors to be extracted, we used the following criteria: (i) eigenvalues > 1, (ii) factor loadings > 0.40, (iii) scree plot and (iv) the total variance explained by the factors. Cronbach’s alpha coefficients were estimated for each factor that emerged from exploratory factor analysis with values > 0.7 indicating acceptable reliability. The tools structure created according to the literature review and respective theory was then validated using confirmatory factor analysis. Adequacy of model fit to the data was evaluated using multiple criteria such as chi-square mean/degree of freedom (CMIN/DF), Tucker Lewis Index (TLI) and the Root Mean Square Error of Approximation (RMSEA). IBM SPSS 21.0 and IBM SPSS AMOS (IBM Corp. Released 2012. IBM SPSS Statistics for Windows, Version 21.0. Armonk, NY: IBM Corp.) were used for statistical analysis.

## Results

The results section is designed so as to present and discuss each step’s results along with the description of the content of the questionnaires.

### Step 1: establishment of the theoretical background/literature review

The literature review conducted towards unraveling measurement tools and dimensions of primary care evaluated, brought into light a wide framework for patient satisfaction components. These components were categorized into several distinct dimensions which seemed to constitute important sources of “satisfaction” and “dissatisfaction” with the primary health care services. Thus, the reported dimensions which constitute the priorities of patients in Greece regarding the primary health care services were:

- **Accessibility** (i.e. ease of accessibility and convenience): It addresses all users for unhindered access of patients to primary health care services such as the amount of time and effort required, the distance to health care services, waiting and servicing times, etc.
- **Technical quality of care and interpersonal (behavioral) aspects**: Technical quality of care concerns the adequacy and ability of health care providers (mainly medical and nursing personnel) and their compliance with the standard procedures of diagnosis and treatment; it includes the perceptions of patients about the skills and abilities of the medical and nursing staff. The interpersonal (behavioral) aspects refer to the quality of care provided to patients and focuses on features that characterize health care providers (mainly medical and nursing personnel) such as kindness, provision of adequate information, interest in the condition of the patient, etc.
- **Continuity of care**: It concerns the systematic provision of health care services by the same health care provider.
- **The physical environment**: It relates to the physical spaces where the health care services are being provided. In particular, space, comfort, cleanliness, clarity of signs to facilitate patients’ proper orientation in the healthcare facilities, etc. are considered to be important sources of satisfaction.

The majority of the studies used self-administered questionnaires, while a wide variation among the used instruments was observed, in terms of questionnaire type. However, almost all studies used the Likert scale with different kind of categories, in order to rate a large number of different experience dimensions. With regard to the studies evaluating patient satisfaction and quality perceptions in Greece, 11 studies conducted during 2005–2016 were identified [11, 24–33]. For the most of these studies, reliability was assured as internal consistency was assessed while several studies conducted a pilot study, and even expert panel feedback was used, to ensure not only internal consistency but also face validity and usability.

Regarding the measurement tools used in patient experience surveys in primary healthcare settings, five questionnaires (out of 15 initially recorded) [34–48] were identified as the most appropriate and relevant to the purposes of this study and were used to develop a relevant, to the national context, patient experience evaluation questionnaire. As a result, following the review of the literature and the measurement tools by the local and international supporting consulting expert team members, the first draft set of question items was developed per type of facility and per dimension of care evaluated.

### Step 2: qualitative study: development of the questionnaire items and establishment of face validity

The two patient focus groups who reviewed the first draft set of questionnaire items, contributed to the development of the preliminary set of questionnaire items [23]. Regarding the Key Stakeholders Consulting Committee’s feedback (first round of interviews) on the first draft set of questionnaire items formulated, accessibility issues, administrative procedures to schedule an appointment with a doctor, continuity of primary care (in terms of collaboration between various types of facilities or professionals, medical history records, etc) and adequacy of supplies were mentioned as dimensions that should be taken into account in the development of the questionnaires. This input, along with the feedback by the supporting expert consulting team and the results from the patient focus groups who reviewed the first set of measurement items, contributed to the development of the preliminary set of questionnaire items. A usability/understandability test was conducted using the focus groups methodology. This enabled reformulating confusing questions and determining a number of problematic items. Problems included problems with clarity, response categories, knowledge and sensitive content, instruction and formatting and a number of situations where items were not applicable. As a result, several questions were rewritten by taking into account participants’ perspectives. Finally, the Key Stakeholders’ Consulting Committee (second round of interviews) revisited the questionnaires and gave their feedback. On the basis of this, the draft questionnaires to be administered in the pilot primary health care facilities were finalized.

### Step 3: quantitative study: pilot testing, establishment of structural validity and estimation of internal consistency

#### Pilot testing

During the pilot testing of the questionnaires, field researchers systematically observed and recorded the various aspects of the collection process and problems encountered, as well as the participants’ responses to the open-ended questions previously designed, keeping in mind the survey’s aim and the goal to pilot test the tools and report back to the local experts team. In general, response rates were relatively high; in almost all facilities, rates were more than 70% (total response rate = 87.3%, 733/839) (the denominator refers to the number of patients asked by the field researchers to complete the survey). Issues regarding organizational aspects and the content of the questionnaires were identified by the field researchers as issues to be further considered and faced upon by the PHC facilities.

#### Assessment of item non-response rates, attributed importance to each item and improvement potential

The non-response rates missing values for the items focusing on nursing staff and other health professionals ranged from 51.9 to 53.3%, while all other items presented low non-responsiveness (ranging from 0.7 to 3.5%). The high percentages of missing values concerning these items can be explained by the role of this staff which is not as well-defined and distinct to the patients as it should be, in the current operating healthcare system, as in many cases, the patients do not meet nurses and/or other health professionals at all. In view of the PHC reform which is due to modify and upgrade their role, placing nurses and other health professionals on a central and distinguished role in the primary health care sector, these items will be relevant to recording patients’ experiences and, therefore, it was decided that these items should be included in the factor analyses that follow. Additionally, low response rates (i.e. high non-response rates) were noted for the items “How was the appointment scheduled?” (26.0%), “How many days did you wait between the appointment and this visit?” (28.0%) and “In case the doctor referred you to another health professional, he/she provided you with adequate information/guidance” (90.0%). Currently, these questionnaire items may indicate no coherent meaning for the participants as the system is fragmented and in fact there is no co-ordination (or a minimum level of co-ordination) and no continuity of services between the various levels of care or sub-systems of the health care system. However, in view of the Primary Health Care System reform foreseen to be implemented by the Ministry of Health, these inefficiencies and gaps are supposed to be confronted. Therefore, it was recommended that these questions should be nevertheless included in the final version of the questionnaire, as there were no matters of non coherence or misunderstandings in the understandability test and the pilot phase of the validation of the questionnaire.

Also, patients were asked to indicate the importance of a statement (this patient values’ questionnaire contained the same questions as the patient experience questionnaire). The approach selected to include the importance items in the survey was to rephrase each experience item into an importance item with the answering categories “Not at all important”, “Slightly important”, “Moderately important”, “Fairly important” and “Very important” on a 5-point Likert scale. A higher score indicated a higher attributed level of importance of a certain care characteristic, according to patients’/users’ perceptions. Based on the users’ responses, the mean importance score for these items ranged from 4.0 to 4.5, indicating high levels of attributed importance to all items.

Finally, the improvement potential was expressed in improvement scores, which were calculated by multiplying the percentage of negative patient experiences for each experience item with the mean importance score attached to that item, with a higher improvement score indicating a higher need / a priority area for improvement.

### Establishment of structural validity and estimation of the internal consistency

The exploratory factor analysis for the 22 items of the “Patients’ Experiences with the care provided by Physicians and other healthcare professionals at Hospital Outpatient Departments” questionnaire (with answers in a five-point Likert scale) is shown in Table 1. Adequacy of the model was assessed with the Bartlett’s test of sphericity and Kaiser-Meyer-Olkin measure. In that case, *p*-value for Bartlett’s test of sphericity was < 0.001 and Kaiser-Meyer-Olkin measure was 0.757 indicating that factor analysis could be conducted. Six factors were extracted and explained 70% of the variability. Cronbach’s alpha for the whole questionnaire was 0.84 indicating very good reliability of the questionnaire items (Cronbach’s alpha coefficient for the four factors was > 0.7 indicating acceptable reliability and only for the last two factors was < 0.70 indicating unacceptable reliability).

**Table 1:** Exploratory factor analysis for the 22 items of the tool

	Factor 1	Factor 2	Factor 3	Factor 4	Factor 5	Factor 6
1. The opening hours are convenient for me					0.50	
2. The facility is close to where I am living or working						0.88
3. It is easy to make an appointment						0.45
4. The doctor asks me about my medical history			0.82			
5. The doctor prescribes to me medication taking into consideration all medications that other doctors have already prescribed			0.80			
6. The doctor asks me about the results of my diagnostic exams incurred in the recent past			0.66			
7. The doctor provides me with advice on how to live healthy				0.66		
8. The doctor clearly explains to me all aspects of my health situation				0.66		
9. The doctor clearly explains to me all aspects of the proposed treatment pathways				0.71		
10. The doctor is polite to me	0.79					
11. The doctor listens to me carefully	0.87					
12. The doctor takes sufficient time to examine me	0.77					
13. The doctor involves me in making decisions about my care and treatment	0.63					
14. The reception staff is helpful	0.84					
15. The navigation within the clinics of this hospital outpatient department is easy	0.54					
16. The waiting room is comfortable					0.76	
17. The hospital outpatient department’s rooms are clean (i.e. clinics, toilets, waiting rooms etc.)					0.76	
18. The clinics of this hospital outpatient department are well-equipped					0.66	
19. The nurses listen to me carefully		0.81				
20. The nurses provides me with advice on how to live healthy		0.73				
21. The nurses are polite to me		0.80				
22. The other health professionals (except doctors and nurses) listen to me carefully		0.84				
Cronbach’s alpha coefficient	0.84	0.82	0.74	0.84	0.64	0.42

Values express factor loadings

Questions 19 to 22 were answered by patients only if they interacted with nurses and other health professionals. So, there was a great number of missing values in questions 19 to 22 since 301 out of 733 patients did not see nurses and other health professionals. The exploratory factor analysis clearly identified items 19, 20, 21 and 22 as a separate factor, despite the increased amount of missing values and concluded to the distinct separate factor of “Quality of care provided by nurses and other health professionals” (see Table 1).

Also, it resulted to the formation of two distinct factors (Factor 3, involving items on continuity and coordination i.e. 4–6 and Factor 4, involving items on comprehensiveness i.e. 7–9). Regarding the remaining items, the exploratory factor analysis did not clearly conclude to distinct factors with conceptual coherence to the theoretical framework and consistent to the dimensions of care identified via the literature review. As a result, and in order to result to more robust and distinct factors that concretely describe patients’ experiences and are coherent with the theoretical framework revealed in the literature review, a subsequent confirmatory factor analysis was decided upon, excluding the items concerning nursing care to avoid the great percentage of missing values. Validation process was resumed by not including these items about nurses and other health professionals in the factor analyses that followed. Therefore, findings from the first exploratory factor analysis suggested that items regarding the Quality of care provided by nurses and other health professionals, Continuity and coordination and Comprehensiveness, respectively, should constitute separate factors added to the final version of the questionnaires.

**Fig. 2:**
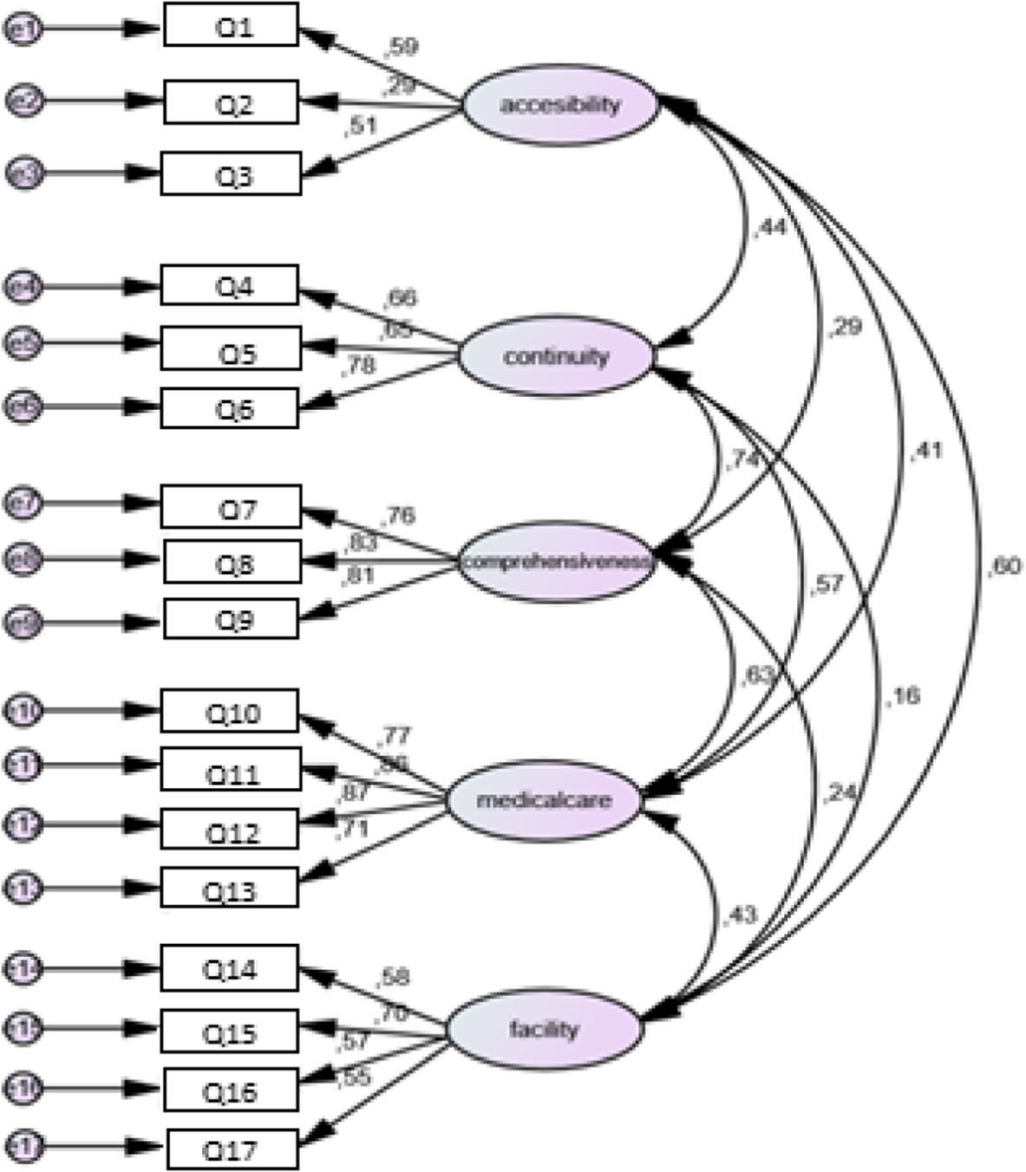
Confirmatory factor analysis

According to the literature review and the respective theory, six factors were created (Table 2) and confirmatory factor analysis was performed in order to check the model (Fig. 2).

**Table 2:** The six factors created according to the literature review and respective theory and also the questionnaire items finally included in each dimension of “Patients’ Experiences with the care provided by Physicians and other healthcare professionals at Hospital Outpatient Departments” questionnaire

	Factor 1	Factor 2	Factor 3	Factor 4	Factor 5	Factor 6
	Accessibility	Continuity and coordination	Compre-hensiveness	Quality of medical care	Facility	Quality of care provided by nurses and other health professionals
1. The opening hours are convenient for me	Χ					
2. The facility is close to where I am living or working	Χ					
3. It is easy to make an appointment	Χ					
4. The doctor asks me about my medical history		Χ				
5. The doctor prescribes to me medication taking into consideration all medications that other doctors have already prescribed		Χ				
6. The doctor asks me about the results of my diagnostic exams incurred in the recent past		Χ				
7. The doctor provides me with advice on how to live healthy			Χ			
8. The doctor clearly explains to me all aspects of my health situation			Χ			
9. The doctor clearly explains to me all aspects of the proposed treatment pathways			Χ			
10. The doctor is polite to me				Χ		
11. The doctor listens to me carefully				Χ		
12. The doctor takes sufficient time to examine me				Χ		
13. The doctor involves me in making decisions about my care and treatment				Χ		
14. The navigation within the clinics of this hospital outpatient department is easy					Χ	
15. The waiting room is comfortable					Χ	
16. The hospital outpatient department’s rooms are clean (i.e. clinics, toilets, waiting rooms etc.)					Χ	
17. The clinics of this hospital outpatient department are well-equipped					Χ	
18. The nurses listen to me carefully						Χ
19. The nurses provides me with advice on how to live healthy						Χ
20. The nurses are polite to me						Χ
21. The other health professionals (except doctors and nurses) listen to me carefully						X
Cronbach’s alpha coefficient	0.43	0.74	0.83	0.87	0.68	0.75

As shown in Fig. 2, confirmatory factor analysis confirmed almost perfectly the presumed theoretical model since standardized regression coefficients between the factors and the respective items were > 0.51, except for item 2 (*p*-value< 0.05 in all cases).

For example, the standardized regression coefficients between the factor “continuity” and items 4, 5 and 6 were 0.67, 0.65 and 0.78, respectively. Also, correlations between factors were low to high (0.19 to 0.74) and *p*-values for all co-variances between factors were < 0.05. Finally, adequacy of model fit to the data is marginally acceptable (*p*-value< 0.001, CMIN/DF = 3.41, TLI = 0.8, CFI = 0.86, PCLOSE< 0.001, RMSEA = 0.09). Regarding the first factor (items 1–3), for item 2 the loading factor was calculated near the acceptable lower limit of 0.30. Nevertheless, item 2 (“The facility is close to where I am living or working”) was decided upon still being included in the final version of the questionnaire, as it represents and mainly highlights a very important aspect of the accessibility dimension of care.

Taking into consideration the factor analysis’ results, 6 factors were identified (Accessibility, Continuity and coordination of care, Comprehensiveness of care, Quality of medical care, Facility amenities and Quality of care provided by nurses and other health professionals) and the items included in each one are shown in Table 2. The factor analyses’ results for the “Patients Experiences with a Specialist at a Health Centre” questionnaire were similar to the ones presented here (see Additional files 1 and 2).

## Discussion

Patient experiences constitute essential key component of the evaluation of the quality of the health care system and health care services. It has been proven a valuable tool for assessing the different dimensions of health care and thus it can provide important information in designing effective health care management strategies.

The measurement tools used in the study to conduct future periodic user experience evaluation surveys in the public primary health care facilities were developed via a thorough set of successive phases. This included literature review, identification of stakeholders’ priority areas, considerations of the local/national circumstances and particularities prevailing in the area of Primary Health Care, the implementation of a focus group methodology and a usability test so as to improve the questionnaires’ content, pilot testing and validating them. In particular, the questionnaires’ face value was established by experts and patient focus groups. They were pilot tested on a subset of PHC facilities and users/patients. The validation process included factor analysis and Cronbach’s alpha calculation. The survey items were consequently revised resulting to the finalisation of the content of the measurement tools/questionnaires. Validating the questionnaires’ items was an essential process that helped to ensure that the questionnaires developed are dependable tools to evaluate PHC services in Greece from the patients’ perspective and experiences.

The domains of care included in the final version of the tool were Accessibility, Continuity and coordination of care, Comprehensiveness of care, Quality of medical care, Quality of care provided by nurses and other health professionals, and Facility amenities. These domains, as represented by the respective experience items per domain, appeared to be rather important by the participants/service recipients and were also indicated by all the involved parties and via the procedures undertaken during the establishment of the face and structural validity of the tools (qualitative and quantitative studies). Also, and in comparison to other tools being used in earlier surveys in Greece, this tool, apart from evaluating experience, it also focused on measuring the importance that patients attach to the various aspects of care, as measured by the experience items. The estimation and consideration of these scores did not only help to select the items for the final survey that matter most to the users/patients during the development phase, but also allowed (and may allow in the future) prioritizing which opportunities for improvement were most important from the patient’s perspective during use and implementation (improvement potential areas). Since importance scores do not appear to vary much over time or between institutions [34], it is considered that it will be proven to be significant for decision makers on an institutional and systemic level to measure these scores in a subset of patients once or periodically, and use the results for a number of years, while assessing patients’ experiences.

Based on the experiences of the patients, along with other evaluation and planning tools, the redesigning of the health care system, and especially the primary health care, may be developed within the framework of continuously improving its quality, maximizing the acceptability of the health care services by their users and strengthening the accessibility to services and equity. This knowledge is important for the primary health care facility managers and health care professionals engaged in this sector, as it will provide them with a valuable insight to the quality of the provided services and help them proceed to meaningful comparisons amongst their counterpart healthcare units, identify best practices and priority areas for potential improvement. Therefore, it is recommended that this evaluation surveys should be used as an on-going evaluation tool in the everyday clinical practice.

Primary health care in Greece has been characterized from many weaknesses including problems of access, continuity, coordination and comprehensiveness of primary care [49]. Fragmented governance; absence of a national quality management infrastructure or routinely used indicators to monitor PHC services; lack of incentives for care providers to improve the quality of care; absence of a gatekeeping system and patient lists; services are not family and community oriented; increased private formal and informal payments; very small number and uneven regional allocation of GPs and nurses are some of the pinpointed shortcomings. Currently, a new Primary Care Plan has been formulated by the Ministry of Health, with implementation envisaged over three years aiming to face the former weaknesses. The establishment of a national, decentralized, community-oriented, network of primary care units, staffed with multidisciplinary teams (doctors, nurses, social workers, etc.), that will be the first contact point within the health system and will operate in an integrated way that will allow the better co-ordination in the provision of care, is among the first priorities of this plan. Towards this direction, the establishment of an evaluation mechanism that will allow identifying best practices as well as prioritizing the opportunities for improvement which are important from the patient’s perspective is very important.

### Study limitations

Our study had several limitations. First of all, a convenience sample for the quantitative study both from primary care units and participants was used which may potentially limit the generalizability of the results. Also, we created self-reported questionnaires and the answers might be affected by response bias. Future studies are needed to further test the questionnaires among various populations to produce more valid results.

## Conclusions

We have developed reliable and valid tools to measure users’ experiences in public Primary Health Care facilities in Greece. These tools could be very useful in examining differences between different types of public Primary Health Care facilities and different populations. Also, these tools could serve as a practical guide for future studies to investigate possible relationships between independent variables (e.g. demographics) and positive or negative experiences.

## Additional files

Additional file 1: Patients’ Experiences with the care provided by Physicians and other healthcare professionals at Hospital Outpatient Departments, PDF (Adobe Acrobat) (PDF 328 kb)

Additional file 2: Patients’ Experiences with the care provided by Specialists at a Health Centre, PDF (Adobe Acrobat) (PDF 319 kb)

## Abbreviations

GP: General Practice
PHC: Primary Health Care
QUALICOPC: Quality and Costs of Primary Care in Europe
TOMYs: Local Primary Care Units
WHO: World Health Organization

## References

[1] World Health Organization (WHO). Health topics: Primary health care. 2018. https://www.who.int/primary-health/en/. Accessed 26 Mar 2019.

[2] Soranz D, Pisco LA (2017). Primary health care reform in the cities of Lisbon and Rio de Janeiro: context, strategies, results, learning and challenges. Cien Saude Colet.

[3] Liseckiene I, Miseviciene I, Dudonis M (2012). Organizational changes in the course of the PHC reform in Lithuania from 1994 to 2010. Health Policy.

[4] Vrachatis DA, Papadopoulos A (2012). Primary health care in Greece: current data and perspectives. Nosileftiki.

[5] Pulvirenti M, McMillan J, Lawn S (2014). Empowerment, patient centred care and self-management. Health Expect.

[6] Ferrer L (2015). Engaging patients, carers and communities for the provision of coordinated/integrated health services: strategies and tools.

[7] Martino SC, Weinick RM, Kanouse DE, Brown JA, Haviland AM, Goldstein E (2013). Reporting CAHPS and HEDIS data by race/ethnicity for Medicare beneficiaries. Health services research.

[8] Faber M, Bosch M, Wollersheim H, Leatherman S, Grol R (2009). Public reporting in health care: how do consumers use quality-of-care information?: a systematic review. Med Care.

[9] Schäfer WL, Boerma WG, Kringos DS, De Maeseneer J, Gress S, Heinemann S (2011). QUALICOPC, a multi-country study evaluating quality, costs and equity in primary care. BMC Fam Pract.

[10] Schäfer WL, Boerma WG, Murante AM, Sixma HJ, Schellevis FG, Groenewegen PP (2015). Assessing the potential of improvement of primary care in 34 countries: a cross-sectional survey. Bull World Health Organ.

[11] Lionis C, Papadakis S, Tatsi C, Bertsias A, Duijker G, Mekouris PB (2017). Informing primary care reform in Greece: patient expectations and experiences (the QUALICOPC study). BMC Health Serv Res.

[12] Ware JE, Davies-Avery A, Stewart AL. The measurement and meaning of patient satisfaction: a review of the literature. Rand Corporation. 1977; https://www.rand.org/pubs/papers/P6036.html. Accessed 26 Mar 2019.

[13] Linder-Pelz SU (1982). Toward a theory of patient satisfaction. Soc Sci Med.

[14] Naidu A (2009). Factors affecting patient satisfaction and healthcare quality. Int J Health Care Quality Assur.

[15] Chow A, Mayer E, Darzi AW, Athanasiou T (2009). Patient reported outcome measures: the importance of patient satisfaction in surgery. Surgery.

[16] LaVela SL, Gallan A (2014). Evaluation and measurement of patient experience. Patient Experience Journal.

[17] Vuori H (1987). Patient satisfaction--an attribute or indicator of the quality of care?. QRB Qual RevBull.

[18] Vuori H (1991). Patient satisfaction—does it matter?. Qual Assur Health Care.

[19] Williams B (1994). Patient satisfaction: a valid concept?. Social Sci Med.

[20] Kotsagiorgi I, Gkeka K (2010). Satisfaction of patients from provided quality of care. Vima Asklipiou.

[21] Coulter A, Fitzpatrick R, Cornwell J. The point of care. Measures of patients’ experience in hospital: purpose, methods and uses. The King’s Fund. 2009. https://www.kingsfund.org.uk/sites/default/files/Point-of-Care-Measures-of-patients-experience-in-hospital-Kings-Fund-July-2009_0.pdf. Accessed 26 Mar 2019.

[22] Chen J Integrated care. Patient reported outcome measures and patient reported experience measures - a rapid scoping review. Agency of Clinical Innovation of New South Wales https://www.aci.health.nsw.gov.au/__data/assets/pdf_file/0009/281979/ACI_Proms_Prems_Report.pdf. Accessed 5 June 2018.

[23] Economou C, Bistaraki A, Galanis P, Konstantakopoulou O, Siskou O, Kaitelidou D. A qualitative approach for the development of a patient experiences questionnaire in primary healthcare settings. *International Journal of Caring Sciences (IJCS). (accepted for publication)*. 2019.

[24] Aletras VH, Papadopoulos EA, Niakas DA (2006). Development and preliminary validation of a Greek-language outpatient satisfaction questionnaire with principal components and multi-trait analyses. BMC health Serv res.

[25] Anagnostopoulos F, Liolios E, Persefonis G, Slater J, Kafetsios K, Niakas D (2012). Physician burnout and patient satisfaction with consultation in primary health care settings: evidence of relationships from a one-with-many design. Journal of clinical psychology in medical settings. J Clin Psychol Med Settings.

[26] Frengidou E, Galanis P, Zafeiropoulou M, Diakoumis G, Papadopoulos R, Papagianni A (2017). User satisfaction with the services provided by the PEDY health unit in Kilkis. Archives of Hellenic Medicine.

[27] Papanikolaou V, Zygiaris S (2014). Service quality perceptions in primary health care centres in Greece. Health Expect.

[28] Pappa E, Kontodimopoulos N, Papadopoulos A, Tountas Y, Niakas D (2013). Investigating unmet health needs in primary health care services in a representative sample of the Greek population. Int J Environ Res Public Health.

[29] Pierrakos G, Yiovannis A, Latou D, Goula A, Pateras J, Sarris M. Measurement of the satisfaction in Greece outpatients departments of public hospitals. In: 4th International Conference in Quantitative and Qualitative Methodologies in the Economic and Administrative Sciences. 2015. http://econferences.teiath.gr/index.php/ICQQMEAS/ICQQMEAS2015/paper/viewFile/171/170. Accessed 5 June 2018.

[30] Pini A, Sarafis P, Malliarou M, Tsounis A, Igoumenidis M, Bamidis P (2014). Assessment of patient satisfaction of the quality of health care provided by outpatient services of an oncology hospital. Glob J Health Sci.

[31] Sbarouni V, Tsimtsiou Z, Symvoulakis E, Kamekis A, Petelos E, Saridaki A (2012). Perceptions of primary care professionals on quality of services in rural Greece: a qualitative study. Rural Remote Health.

[32] Matziou V, Servitzoglou M, Vlahioti E, Deli H, Matziou T, Megapanou E (2013). The opinion of Greek parents on the advantages and disadvantages of the outpatient pediatric oncology setting. Europ J Oncol Nurs.

[33] Raftopoulos V (2010). Assessment of users' expectations, perceived quality and satisfaction with primary care in Greece. International journal of caring sciences.

[34] Schäfer WL, Boerma WG, Kringos DS, De Ryck E, Greß S, Heinemann S (2013). (2013). Measures of quality, costs and equity in primary health care: instruments developed to analyse and compare primary health care in 35 countries. Qual Prim Care.

[35] Keller S, O'malley AJ, Hays RD, Matthew RA, Zaslavsky AM, Hepner KA (2005). (2005). Methods used to streamline the CAHPS hospital survey. Health Serv Res.

[36] Pettersen KI, Veenstra M, Guldvog B, Kolstad A (2004). The patient experiences questionnaire: development, validity and reliability. Int J Qual Health Care.

[37] Jenkinson C, Coulter A, Bruster S (2002). (2002). The picker patient experience questionnaire: development and validation using data from in-patient surveys in five countries. Int J Qual Health Care.

[38] Wilde B, Larsson G, Larsson M, Starrin B (1994). Quality of care: development of a patient-centred questionnaire based on a grounded theory model. Scand J Caring Sci.

[39] Larsson BW, Larsson G (2002). (2002). Development of a short form of the quality from the Patient's perspective (QPP) questionnaire. J Clin Nurs.

[40] Boyd J The 2006 inpatients importance study. The acute co-ordination centre for the NHS acute patient survey programme. Picker Institute Europe. 2007. http://www.nhssurveys.org/Filestore/documents/Findings_and_development_of_the_2006_Inpatients_Importance_study_final.pdf. Accessed 5 June 2018.

[41] Scottish Government. Scottish inpatient patient experience survey. 2012. https://www.gov.scot/publications/scottish-inpatient-patient-experience-survey-2012-volume-1-national-results/pages/2/. Accessed 26 Mar 2019.

[42] Wong E, Coulter A, Hewitson P, Cheung A, Yam C, Lui S. Patient Experience and Satisfaction with Inpatinet Service: Development of Shoert Form Survey Instrument Measuring the Core Aspect of Inpatient Experience. PLoSone. 2015;10(4):e0122299. https://www.ncbi.nlm.nih.gov/pmc/articles/PMC4393260/. Accessed 26 Mar 2019. 10.1371/journal.pone.0122299 PMC4393260 25860775

[43] Oltedal S, Garratt A, Bjertnæs Ø, Bjørnsdottìr M, Freil M, Sachs M (2007). The NORPEQ patient experiences questionnaire: data quality, internal consistency and validity following a Norwegian inpatient survey. Scand J Public Health.

[44] Webster TR, Mantopoulos J, Jackson E, Cole-Lewis H, Kidane L, Kebede S (2011). A brief questionnaire for assessing patient healthcare experiences in low-income settings. Int J Qual Health Care.

[45] Rao KD, Peters DH, Bandeen-Roche K (2006). Towards patient-centered health services in India-a scale to measure patient perceptions of quality. Int J Qual Health C.

[46] Teale EA, Young JB (2015). A patient reported experience measure (PREM) for use by older people in community services. Age Ageing.

[47] Triemstra M, Winters S, Kool RB, Wiegers TA (2010). Measuring client experiences in longterm care in the Netherlands: a pilot study with the consumer quality index Longterm care. BMC Health Serv Res.

[48] Glasgow RE, Wagner EH, Schaefer J, Mahoney LD, Reid RJ, Greene SM (2005). Development and validation of the patient assessment of chronic illness care (PACIC). Med Care.

[49] Groenewegen P, Jurgutis A (2013). A future for primary care for the Greek population. Qual Prim Care.

