# Supplementary material for: Development and validation of measurement tools for user experience evaluation surveys in the public primary healthcare facilities in Greece: a mixed methods study
Source: BMC Fam Pract. 2019 Apr 2;20:49. doi: 10.1186/s12875-019-0935-6 (PMC6444824; doi:10.1186/s12875-019-0935-6)
Supplement: Supplementary file 2 — Patients’ Experiences with the care provided by Specialists at a Health Centre, PDF (Adobe Acrobat) (PDF 319 kb) [file 12875_2019_935_MOESM2_ESM.pdf]

Patients' Experiences with the care provided by Specialists at a Health Centre

1. What is your gender?
- ☐ Male
- ☐ Female

2. What is your birth year? ☐☐☐☐

3. What is your nationality?
- ☐ Greek
- ☐ Other (Please define: ……………………….........................…..………….)

4. What is the highest level of education that you have achieved?
- ☐ I never finished Primary school
- ☐ Primary school
- ☐ Secondary school
- ☐ High School
- ☐ After High School education
- ☐ Higher education

5. Regarding your insurance status: (You can choose more than one)
- ☐ I am insured at EOPYY or other social/public security fund
- ☐ I am insured at a private insurance company
- ☐ I am not insured

6. Are you disabled more than 67%?
- ☐ Yes
- ☐ No

7. How would you describe your health in general?
- ☐ Excellent
- ☐ Very good
- ☐ Good
- ☐ Moderate
- ☐ Bad

8. Have you suffered from a chronic disease (i.e. a disease that you are suffering from for more than a year)?
- ☐ No, none
- ☐ Yes, one
- ☐ Yes, two
- ☐ Yes, three or more
- ☐ I don't know

9. In case you suffer from a chronic disease, please indicate it. (You can choose more than one)
- ☐ Cardiovascular disease (i.e. stroke, heart failure etc.)

- ☐ Respiratory disease (i.e. asthma, chronic obstructive pulmonary disease etc.)
- ☐ Autoimmune disease (i.e. ulcerous colitis, multiple sclerosis, rheumatoid arthritis etc.)
- ☐ Thyroid disease (i.e. hypothyroidism, hyperthyroidism, Hashimoto disease etc.)
- ☐ Cancer
- ☐ Diabetes mellitus
- ☐ Kidney disease
- ☐ Other (Please define: ……………………………………………………………)

10. Over the past six months, how often did you visit or consulted this facility?

- ☐ Never
- ☐ Once
- ☐ 2-4 times
- ☐ ≥ 5 times
- ☐ I don’t know/I don’t remember

11. How did you visit this facility today?

- ☐ With a physician's referral
- ☐ Without a physician's referral

12. Which of the following professionals did you visit today?

GP	☐	Dietician	☐
Internist	☐	Dentist	☐
Pediatrician	☐	Nurse	☐
Radiologist	☐	Health visitor	☐
Microbiologist	☐	Midwife	☐
Cardiologist	☐	Physiotherapist	☐
Dermatologist	☐	Occupational therapist	☐
Otolaryngologist	☐	Speech therapist	☐
Ophthalmologist	☐	Social worker	☐
Pulmonologist	☐	Psychologist	☐
Psychiatrist	☐	Other	☐
Endocrinologist	☐	Please define::…………………………………………………..	

13. What is the reason for your visit to this facility today? (You can choose more than one)

- ☐ Sick/Unwell
- ☐ Scheduled follow-up visit/ Scheduled medical check-up
- ☐ Prescription of medications
- ☐ Prescription of lab exams (diagnostic imaging, blood or urine tests, etc)
- ☐ Referral from another physician/primary health care facility
- ☐ Medical certificate
- ☐ Advisory support (i.e. childbirth education)
- ☐ Other (Please define: ……………………………………………………………)

14. Did you make an appointment for your visit to this facility?

- ☐ Yes
- ☐ No *(in case you checked «No», please move to question 17)*

15. How was the appointment scheduled?

- ☐ By visiting the reception
- ☐ By phone at the reception
- ☐ By phone in 5-digit calling number
- ☐ I arranged it directly with the doctor
- ☐ Via Internet
- ☐ I don’t know, since somebody else scheduled the appointment on my behalf

16. How many days did you wait between the appointment and this visit?

- ☐ I made the appointment earlier today
- ☐ I made the appointment yesterday
- ☐ I waited less than a week
- ☐ I waited from 1 week to 1 month
- ☐ I waited more than 1 month
- ☐ I don’t know/I don’t remember

17. How long did you wait today for the completion of the administrative procedures, before the consultation (i.e. waiting queue in reception)?

- ☐ Less than 15 minutes
- ☐ 15-30 minutes
- ☐ 31-60 minutes
- ☐ More than 60 minutes
- ☐ I don’t know/I don’t remember

18. How long did you wait today between completing the administrative procedures and the consultation?

- ☐ Less than 15 minutes
- ☐ 15-30 minutes
- ☐ 31-60 minutes
- ☐ More than 60 minutes
- ☐ I don’t know/I don’t remember

19. The doctor:

- ☐ did not refer me to someone else (*in case you checked this box, please move directly to question* 21)
- ☐ referred me to another doctor in this facility
- ☐ referred me to a hospital
- ☐ referred me for lab exams in this facility
- ☐ referred me for lab exams outside this facility

20. In case the doctor referred you to another health professional, he/she provided you with adequate information/guidance (i.e. working hours, accessibility, and contact details).

*Please rate from 1 to 5 (where **1** stands for **I totally disagree** and **5** stands for **I totally agree**)*

1	2	3	4	5
☐	☐	☐	☐	☐

21. Today, did you visit a nurse or another health professional in this facility without visiting a doctor?

☐ Yes

☐ No

Think about your visit today. Do you agree with the following?

*Please rate from 1 to 5 (where **1** stands for **I totally disagree** and **5** stands for **I totally agree**)*

	**I totally disagree 1**	**I disagree 2**	**Neither agree nor disagree 3**	**I agree 4**	**I totally agree 5**
**22.** The opening hours are convenient for me	☐	☐	☐	☐	☐
**23.** The facility is close to where I am living or working	☐	☐	☐	☐	☐
**24.** It is easy to make an appointment	☐	☐	☐	☐	☐
**25.** The doctor asks me about my medical history	☐	☐	☐	☐	☐
**26.** The doctor prescribes to me medication taking into consideration all medications that other doctors have already prescribed	☐	☐	☐	☐	☐
**27.** The doctor asks me about the results of my diagnostic exams incurred in the recent past	☐	☐	☐	☐	☐
**28.** The doctor provides me with advice on how to live healthy (i.e. about physical exercise, smoking, food, drinking, medication, sleeping habits etc)	☐	☐	☐	☐	☐
**29.** The doctor clearly explains to me all aspects of my health situation	☐	☐	☐	☐	☐
**30.** The specialist clearly explains to me all aspects of the proposed treatment pathways	☐	☐	☐	☐	☐
**31.** The doctor is polite to me	☐	☐	☐	☐	☐
**32.** The doctor listens to me carefully	☐	☐	☐	☐	☐
**33.** The doctor takes sufficient time to examine me	☐	☐	☐	☐	☐
**34.** The doctor involves me in making decisions about my care and treatment	☐	☐	☐	☐	☐
**35.** It is easy to orient myself within the premises and areas/rooms of this facility	☐	☐	☐	☐	☐
**36.** The waiting area is convenient	☐	☐	☐	☐	☐
**37.** The areas (i.e. the physicians' offices, toilets, waiting areas, etc) of this facility are clean	☐	☐	☐	☐	☐
**In case you DID not see today nurses or other health professionals please move to question 43**					
**38.** The nurses listen to me carefully	☐	☐	☐	☐	☐
**39.** The nurses provides me with advice on how to live healthy	☐	☐	☐	☐	☐
**40.** The nurses are polite to me	☐	☐	☐	☐	☐
**41.** The other health professionals (except doctors and nurses) listen to me carefully	☐	☐	☐	☐	☐

42. On a scale of 0-10, would you recommend the doctor to your friends and/or relatives?
*Please rate from 0 to 10 (where **0** stands for **Definitely not** and **10** stands for **Certainly yes**)*

0	1	2	3	4	5	6	7	8	9	10
☐	☐	☐	☐	☐	☐	☐	☐	☐	☐	☐

43. On a scale of 0-10, would you recommend this facility to your friends and/or relatives?
*Please rate from 0 to 10 (where **0** stands for **Definitely not** and **10** stands for **Certainly yes**)*

0	1	2	3	4	5	6	7	8	9	10
☐	☐	☐	☐	☐	☐	☐	☐	☐	☐	☐

Please note that this section of the questionnaire refers to the **IMPORTANCE of the previously asked items** used in this questionnaire. Rate them according to their importance for you.

	Not at all Important	Slightly Important	Moderately Important	Fairly important	Very important
**44.** Waiting time between the appointment and this visit	☐	☐	☐	☐	☐
**45.** Waiting time for the completion of the administrative procedures (i.e. waiting queue in the reception etc.)	☐	☐	☐	☐	☐
**46.** Waiting time between completing the administrative procedures and the consultation	☐	☐	☐	☐	☐
**47.** In case the doctor referred you to another health professional, he/she provided you with adequate information/guidance (i.e. working hours, accessibility, and contact details)	☐	☐	☐	☐	☐
**48.** The opening hours are convenient for me	☐	☐	☐	☐	☐
**49.** The facility is close to where I am living or working	☐	☐	☐	☐	☐
**50.** It is easy to make an appointment	☐	☐	☐	☐	☐
**51.** The doctor asks me about my medical history	☐	☐	☐	☐	☐
**52.** The doctor prescribes to me medication taking into consideration all medications that other doctors have already prescribed	☐	☐	☐	☐	☐
**53.** The doctor asks me about the results of my diagnostic exams incurred in the recent past	☐	☐	☐	☐	☐
**54.** The doctor provides me with advice on how to live healthy (i.e. about physical exercise, smoking, food, drinking, medication, sleeping habits etc)	☐	☐	☐	☐	☐
**55.** The doctor clearly explains to me all aspects of my health situation	☐	☐	☐	☐	☐
**56.** The doctor clearly explains to me all aspects of the proposed treatment pathways	☐	☐	☐	☐	☐
**57.** The doctor is polite to me	☐	☐	☐	☐	☐
**58.** The doctor listens to me carefully	☐	☐	☐	☐	☐
**59.** The doctor takes sufficient time to examine me	☐	☐	☐	☐	☐
**60.** The doctor involves me in making decisions about my care and treatment	☐	☐	☐	☐	☐
**61.** The staff at the reception is polite to me	☐	☐	☐	☐	☐
**62.** It is easy to orient myself within the premises and areas/rooms of this facility	☐	☐	☐	☐	☐
**63.** The waiting area is convenient	☐	☐	☐	☐	☐
**64.** The areas (i.e. the physicians' offices, toilets, waiting areas, etc) of this facility are clean	☐	☐	☐	☐	☐

	Not at all Important	Slightly Important	Moderately Important	Fairly important	Very important
**65.** The nurses listen to me carefully	☐	☐	☐	☐	☐
**66.** The nurses provide me with advice on how to live healthy	☐	☐	☐	☐	☐
**67.** The nurses are polite to me	☐	☐	☐	☐	☐
**68.** The other health professionals (except doctors and nurses) listen to me carefully	☐	☐	☐	☐	☐

69. What gave you positive impressions during your visit today?

70. According to you what could the doctor or/and the other health professionals improve?

71. According to you what could be improved in this facility?

Thanks a lot for your participation and your time!
